# Draft Genome Sequences of Three *Escherichia coli* Strains Investigated for the Effects of Lysogeny on Niche Diversification

**DOI:** 10.1128/genomeA.00955-14

**Published:** 2014-09-25

**Authors:** Jennifer Y. H. Lai, Hao Zhang, Miranda H. Y. Chiang, Min Yu, Rui Zhang, Stanley C. K. Lau

**Affiliations:** a Division of Life Science, the Hong Kong University of Science and Technology, Hong Kong; b State Key Laboratory of Marine Environmental Science, Xiamen University, Xiamen, China

## Abstract

During the course of investigating the effects of lysogeny on niche diversification of *Escherichia coli*, we used the temperate phages induced from one *E. coli* strain to infect another and created an isogenic lysogen of the latter. The draft genome sequences of the three *E. coli* strains are reported herein.

## GENOME ANNOUNCEMENT

Prophages have contributed 26% of strain-specific genes to the pangenome currently identified for commensal and pathogenic *Escherichia coli* (1). Through the addition of new genes and the regulation of host genes, prophages have profound effects on the metabolism, physiology, and hence ecological niche of *E. coli* (2–4). In our investigation of lysogeny as a mechanism mediating the survival of *E. coli* outside animal hosts, we isolated *E. coli* E1140 from marine sediment and E455 from pig feces. Prophages associated with E1140 were induced to release phage particles using mitomycin C (5, 6). The induced phages were taken to infect E455. An isogenic lysogen, namely, E455L was thus created.

The draft genome sequences of the three strains were obtained using 300-bp insert pair-end libraries on the Illumina HiSeq2000 system and 10 kb-insert single molecule, real-time (SMRT) technology on the PacBio RS system. The reads were *de novo* assembled manually into contigs using CLC Genomics Workbench version 6.5.1 (CLC bio, USA) and the Celera Assembler version 8 (7). We obtained 11 contigs of 1,804 to 2,924,481 bp in size (*N*_50_ = 2,924,481 bp) with a total size of 5,111,617 bp for E1140, 6 contigs of 5,215 to 3,090,951 bp in size (*N*_50_ = 3,090,951 bp) with a total size of 4,587,712 bp for E455, and 15 contigs of 5,720 to 2,472,449 bp in size (*N*_50_ = 2,472,449 bp) with a total size of 4,592,228 bp for E455L.

Using the Glimmer2 (8), the numbers of protein coding sequences predicted were 4,989 for E1140, 4,353 for E455, and 4,390 for E455L. The prophages in the draft genome sequences were identified using the PHAge Search Tool (PHAST) (9). E455 was found to carry two prophages. E455L carried not only the two prophages of E455, but also an extra P2 prophage that was 30,983 bp in size and comprised of 37 phage genes and 6 genes of unknown functions. E1140 carried seven prophages, including a P2 that was identical to the one found in E455L. These results suggest that E455 had received a P2 prophage of E1140 during lysogenic infection, resulting in an isogenic lysogen E455L.

### Nucleotide sequence accession numbers.

These whole-genome shotgun projects have been deposited at DDBJ/EMBL/GenBank under the accession numbers JEND00000000, JENE00000000, and JDFU00000000 for *E. coli* strains E455, E455L, and E1140, respectively. The versions described in this paper are JEND02000000, JENE03000000, and JDFU02000000 for E455, E455L, and E1140, respectively.
